# Why Are the Benefits of Increased Resources Not Impacting the Risk of HIV Infection for High SES Women in Cameroon?

**DOI:** 10.1371/journal.pone.0100507

**Published:** 2014-06-26

**Authors:** Joyce N. Mumah, Douglas Jackson-Smith

**Affiliations:** 1 African Population and Health Research Center, Population Dynamics and Reproductive Health Program, Nairobi, Kenya; 2 Utah State University, Department of Sociology, Social Work and Anthropology, Logan, Utah, United States of America; UCL Institute of Child Health, University College London, United Kingdom

## Abstract

**Background:**

Despite evidence of a positive SES-HIV gradient in some SSA countries, researchers and policy-makers frequently assume that a range of protective interventions – increasing awareness of mechanisms of HIV transmission, techniques for prevention, greater access to health care facilities, and greater availability of condoms – will reduce the likelihood of contracting HIV, even among higher SES populations. We therefore explore the relationships between SES and these intervening behaviors to illuminate the complex factors that link SES and HIV among women in Cameroon.

**Methods:**

We use bivariate and multivariate statistical analysis to examine patterns among the 5, 155 women aged 15–49 who participated in the 2004 CDHS.

**Results:**

The results show a strong pattern where higher SES women have greater access to and use of health care facilities, higher levels of condom use, more HIV knowledge, and command higher power within their relationships, yet also have higher rates of HIV. These traditionally protective factors appear to be offset by riskier sexual behaviors on the part of women with increased resources, most notably longer years of premarital sexual experience, multiple partners in last 12 months, and sexual encounters outside of relationship. Multivariate analyses suggests net of the effect of other factors, women who command higher decision-making power, have greater access to health care, more negative attitudes toward wife beating, longer years of premarital sexual exposure, and partners with professional/white collar jobs (characteristics associated with rising SES) had higher odds of testing positive for HIV.

**Conclusion:**

Results show that higher riskier sexual practices on part of high SES women offset benefits that may have accrued from their increased access to resources. The results suggest that traditional approaches to HIV prevention which rely on poverty reduction, improving access to health care, improving HIV knowledge, and boosting women’s social and economic power may be insufficient to address other drivers of HIV infection among women in SSA.

## Background

The sub-Saharan (SSA) HIV epidemic presents patterns that challenge conventional understandings of the relationships between socioeconomic status (SES) and health. The notion that poverty was the underlying factor increasing susceptibility to HIV infection had become one of the main messages sent out by the global health field [1]. It has often been assumed that Africa’s status as the continent with the highest HIV infection rates is linked to its endemic high poverty and a corresponding lack of access to adequate health care resources [2]. Poverty reduction strategies were therefore touted as the most viable long-term option in the fight against the disease [3]. In SSA, high SES individuals have higher levels of formal education, greater exposure to health education messages and mass media, increased access to health care, and better access to condoms, which should all lower their risk for contracting HIV [4].

However, a growing number of studies show that wealthier individuals (irrespective of gender), wealthier regions (urban areas), and wealthier countries within SSA often display the highest levels of HIV infection [5]–[15]. In a study of eight African countries, Mishra and colleagues [12], provide evidence of a positive association between household wealth and HIV prevalence. Similarly, a review of thirty-five studies on SES and HIV status in SSA, found ten cross sectional studies and one cohort study that described a positive association, between high SES and HIV infection [15]. Theories for why SES and HIV are positively related often point to social and cultural mechanisms, especially lifestyles associated with increased income (such as travel and access to multiple partners) which are associated with increased vulnerability to HIV infection [5]. Of primary interest therefore, are patterns of risky sexual behavior that are positively linked to an individual’s wealth, including the number of multiple and non-marital sexual partners, and premarital sexual intercourse. Mumah and Jackson-Smith [16], in a recent study found that the length of premarital sexual activity among high SES women significantly increases their vulnerability to HIV, because high SES women are more likely to delay first marriage, yet be sexually active. For men, level of education and income increases the probability individuals will have non-regular sexual partners, which is known to also increase exposure to STDs including HIV [17]–[18]. Kongnyuy and colleagues [17], found that wealthy men in Cameroon had higher rates of HIV infection, which in part was explained by their riskier sexual behaviors including earlier sexual debut, multiple concurrent and lifetime partners, and limited use of condoms with partners other than wives or cohabiting partners.

Similar patterns are found for high SES women. One recent study argues that it is the ‘pursuit of modernity’ that explains female risky sexual behaviors in SSA [18]. The authors found evidence from Kenya and South Africa that materialism and pursuit of consumer goods such as cars, cash and cell phones drives transactional sex, rather than absolute poverty [18]–[19]. Fox [5], argues therefore that for women, having multiple partners can be a mechanism for achieving upward mobility, while for men, multiple partnership is an avenue to validate ‘sexual prowess and social status’. Similarly, numerous studies indicate that the direction of transmission of HIV among migrants is not limited to transmission from migrant males to their wives, but also from the non-migrating wives to their spouses, because they had other sexual partners while their spouses were away [20].

As in the case with wealth, higher levels of formal education are often associated with increased risk of HIV, particularly in Eastern and Southern Africa [10], [21]–[22]. One study found that those with basic primary education had twice the odds of being HIV-positive as those with no education [10]. Studies that find a positive association between wealth and HIV often do not find the same pattern with education, suggesting that SES-HIV linkages are complex [22]–[25]. Moreover, controlling for other individual characteristics can reduce or eliminate statistical associations between HIV status and education levels [26].

Despite evidence of a positive SES-HIV gradient, researchers and policy-makers frequently assume that a range of protective interventions – increasing awareness of mechanisms of HIV transmission and techniques for prevention, greater access to health care facilities (including HIV testing and counseling clinics), and greater availability of condoms – will reduce the likelihood of contracting HIV [27]–[28], even among higher SES populations [29]. Similarly, researchers have called for empowering women to have more control over sexual decision-making through socioeconomic development [6]. The presumed mechanism for protection is a change in behavior that could place a person at risk for contracting HIV.

**Figure 1:**
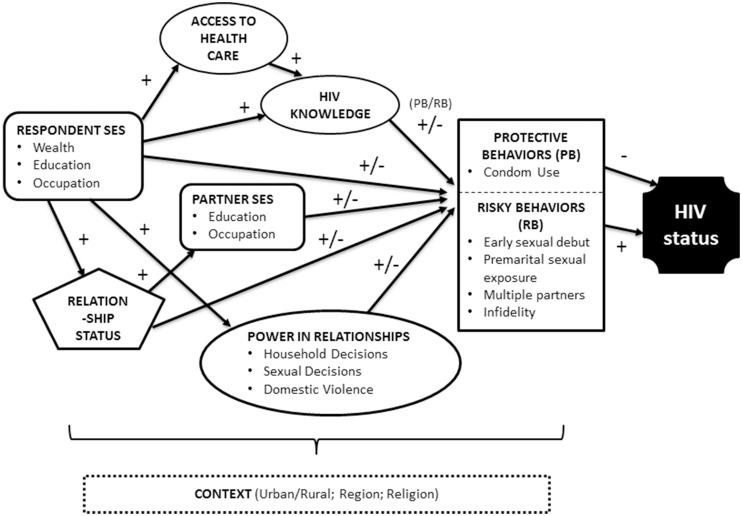
Theoretical Model Linking SES and HIV Status.

In this paper, we examine evidence from a nationally representative survey of Cameroonian women and explore how SES is related to HIV status. Previously published research documented that higher SES women are much more likely to contract HIV than their low SES counterparts [22], [24]–[26], [30]–[31]. Our analysis accounts for three core sets of intervening variables (access to health care, HIV knowledge, and power in relationships), and assesses the key role of risky and protective sexual behaviors in determining HIV status. We also account for the effects of partner SES, depending on the marital status of the woman, and control for several other contextual characteristics. A graphic representation of our theoretical model, with illustrations of the hypothesized direction of relationships can be found in Figure 1.

## Data and Methods

### Data

The 2004 Cameroon Demographic and Health Survey (CDHS), is a nationally representative survey involving residents aged 15–49. The DHS uses a multi-layer protocol review system to obtain ethical review in the United States and host country before data collection begins. The survey and the protocol for blood specimen collection and analysis were approved by the Institutional Review Board for ORC Macro, Calverton, USA. In Cameroon, the National Ethics Committee in the Ministry of Health reviewed the protocol and provided ethical clearance and approval. Written informed consent, which was vetted and approved by the National Ethics Committee in Ministry of Health, was gotten from all survey participants prior to participation and all information was collected confidentially. The informed consent forms were translated to the appropriate local language before being administered to participants.

The CDHS uses a multi-stage complex cluster sampling methodology with response rates well in excess of 90 percent [32]–[34]. The CDHS survey provides information on a variety of topics including HIV test results for nearly all respondents, determined via voluntary blood samples. The CDHS included 5, 155 female respondents in the sample, with conclusive test results for all but one case. The ability to link HIV testing with individual characteristics provides a unique opportunity to assess factors associated with HIV infection. Analysis in this study was run using both weighted and unweighted data. The results shown in this paper uses the unweighted sample data because there were no meaningful or substantive differences between results estimated from the weighted and unweighted samples.

#### Measures

Socioeconomic status (SES) is captured both at the individual-level (respondent *educational attainment* and *occupational status*) and household-level (based on a *wealth index)*. Female SES has been linked both to the timing and nature of marriage and to the SES of her partner, therefore the impact of partner’s SES on a woman’s HIV risk is mediated by her marital status/relationship status and history. In the analysis, dummy variables for currently married, never married, cohabiting and early marriage are used.

The first two sets of intervening variables reflect better access to health care and knowledge about HIV, which we expect to increase with higher levels of SES [29]. Access to health care is captured by indicators of respondents’ awareness and use of Centre de Dépistage volontaire clinics (CPDV; voluntary HIV counseling and testing centers), whether the respondent visited any health facility in the last 12 months, and self-reports of barriers to accessing local health clinics (including *transportation, distance, and knowledge of where to go).* Knowledge of HIV infection and prevention mechanisms was measured by constructing an additive scale using 15 knowledge items in the CDHS [35]. The scale met conventional standards for reliability (Cronbach’s alpha = 0.738) and ranges in value from 0–15. A woman’s ability to access health care will influence her knowledge about HIV. In turn, a woman’s knowledge of HIV and prevention methods is presumed to influence her behaviors which could be risky or protective, thereby determining her HIV status.

The third set of intervening variables reflects a woman’s decision-making power in her relationships. Power in relationships is captured in scale constructed from questions about household decision making (5 items; Cronbach’s alpha = 0.827), attitudes toward wife beating (5 items; Cronbach’s alpha = 0.797), and perceptions of a woman’s power in sexual decision-making (4 items, Cronbach’s alpha = 0.579). SES is theoretically linked to a woman’s power in her sexual relationships, which in turn should influence the behaviors of both women and their partners. We theorize here that the more power a woman commands in her relationship the less risky will be her behaviors (and vice versa).

Our theoretical model (Figure 1) captures the fact that HIV status is most directly affected by sexual behaviors and the influence of SES, access to health care, HIV knowledge and power should manifest as differences in levels of these behaviors. Female sexual behavior is captured in our analysis via both female protective sexual behaviors (three indicators of *condom use)*, and female risky sexual behaviors *(e.g. length of premarital sexual experience, having sex with a person other than partner, and having sex with more than one partner in the last 12 months).*

Finally, our analysis controls for other contextual factors including place of residence, region and religion. Dummy variables for rural/urban residence and cultural regions are used because there are large regional and urban/rural differences within the country and a study of SES and risk of HIV needs to take these contextual factors into consideration. Due to large number of ethnic categories with some having very few cases, a collapsed version of region, rather than ethnicity is used in our analyses. Kuate-Defo [36], posits that the more than 200 ethnic groups in Cameroon fall into four major cultural regions of the country: Center, South, and East; Littoral and South West; West and North West; and Nord, Adamaoua and Extreme Nord. This collapsed version of regions is used as a proxy for ethnicity. Because religious affiliations are not perfectly correlated with region, dummy variables for the respondent’s personal religious affiliation: Catholic, Protestant, Muslim and Other are included.

#### Analyses

Bivariate analyses are first utilized to illustrate the simple associations among women’s SES status, each of the intervening variables, and HIV status. We begin by describing the relationship between HIV and all the other variables to highlight the factors most associated with the prevalence of HIV infection among women in Cameroon. Second, we examine the relationships between SES and the intervening variables to see if SES is associated with the expected intervening variables in ways that are expected to increase or decrease HIV risk. Third, we look at the link between access to health care, HIV knowledge, and indicators of sexual behavior. Three types of bivariate analyses are used: cross tabulations, ANOVA-tests, and *t*-tests. Chi-square and Fishers’ Exact tests are used to determine statistical relationship between the categorical variables analyzed in cross tabulations. Strength of association was assessed using Cramer’s V and gamma. We then estimate two multivariate binary logistic regression models to contrast the effects of women’s SES status on HIV with and without the presence of intervening variables. The full model also estimates the relative contribution of access, knowledge, and power to explaining the positive bivariate SES-HIV relationship. The multivariate models use 4,916 cases because 239 cases were missing at least one core variable used in the analyses. The overall fit of the model was assessed using the negative log-likelihood (−2LL) and the Homer and Lemeshow statistic (H&L), while the explanatory adequacy of the model were assessed using the Cox & Snell and Nagelkerke R-Squares. This study utilized IBM SPSS version 19 to manage and analyse the data.

## Results

### Bivariate analyses

#### Relationship between Core Variables and HIV Status

As noted above, the CDHS provides a rare opportunity to combine information about the characteristics of large representative samples of adults with actual HIV blood test results. Table 1 shows the proportion of women in the CDHS sample and the incidence of HIV by various indicators of SES. The results clearly show that household wealth, educational attainment, and occupational status are significantly and positively related to HIV status among women in Cameroon. A woman’s partners’ SES is also associated with her HIV status. Among the 23 percent of women with no current partner, HIV rates are relatively low (3.5%). However, the incidence of HIV among women increases systematically with her partners’ educational attainment and occupational status.

**Table 1:** Prevalence of HIV Infection by SES and Other Respondent Characteristics, Women Respondents, 2004 CDHS.

Characteristic	% of Womenin the CDHS	% HIV Positive	*Sign.*
**Socioeconomic Status**			
*Household Wealth Index*			
Low	35.2	4.1	***
Medium	22.3	7.6	
High	42.5	8.5	
*Respondent Educational Attainment*			
No Education	19.8	3.9	***
Primary Education	41.1	7.0	
Secondary or More	39.1	8.0	
*Respondent Occupation*			
Unemployed	37.5	5.8	***
Agriculture	34.8	5.7	
Manual/Domestic	23.4	9.7	
Professional/White Collar	4.4	8.0	
**Partner’s Socioeconomic Status**			
*Partner’s Educational Attainment*			
No Partner	22.8	35.0	***
No Education	15.7	3.5	
Primary Education	23.3	7.3	
Secondary Education	28.2	9.2	
Higher Education	4.8	12.8	
Not Reported	4.9	10.0	
*Partner’s Occupation*			
No Partner	22.8	3.5	***
Unemployed	2.1	4.7	
Agriculture	32.4	4.3	
Manual/Domestic	20.6	10.0	
Professional/White Collar	21.4	10.8	
**Access to Health Care**			
*Access to CPDV*			
Not Heard of CPDV	74.2	6.5	***
Heard but not been	22.9	6.9	
Been to a CPDV	2.9	14.1	
*Visited a Health Facility last 12 months*			
No	47.5	5.5	***
Yes	52.5	7.9	
*Faced Barriers to Medical Care*			
No Barriers	52.9	8.0	***
Faced at least 1 barrier	13.9	5.5	
Faced at least 2 barriers	23.6	5.4	
Faced at least 3 barriers	9.5	4.9	
**Protective Behaviors**			
*Ever Used a Condom*			
No	66.5	5.3	***
Yes	33.5	9.7	
*Condom Use Last Sexual Intercourse*			
No	88.9	6.5	*n.s.*
Yes	11.1	7.5	
*Condom Use Currently*			
No	90.6	6.7	*n.s.*
Yes	9.4	7.7	
**Risky Behaviors**			
*Respondent Used Alcohol at Last Sex*			
No	94.7	7.2	***
Yes	14.8	10.0	
*Sex Outside Relationship*			
No	90.5	6.3	***
Yes	9.5	10.9	
*Multiple Partners*			
No	93.3	6.4	***
Yes	6.7	11.0	
*Early First Sexual experience*			
*(under 15)*			
No	65.0	5.2	**
Yes	35.0	7.6	
*Overall HIV prevalence among women (N = 5154)*	349.0	6.6	

n.s. = not statistically significant (p>0.05).

***p<0.001 for Chi-Square (Fischer’s Exact Test for 2×2 tables).

The relationship between access to health care, sexual behaviors, and HIV rates among Cameroonian women is also shown in Table 1. There is a strong pattern where women with greater access to and use of health care facilities have higher rates of HIV. It is unlikely that the use of health care resources causes HIV infection, so this counterintuitive finding may reflect a pattern in which HIV-positive women are more likely to go to health care providers because they suffer from symptoms of the disease. Alternatively, women living in more remote rural areas have less access to health care, but also face a much lower overall background rate of population HIV infection.

A similar pattern is also seen in Table 1 where women who have higher rates of condom use are also more likely to test positive for the HIV virus. Again, it is possible that condom use is caused by positive HIV status (rather than vice versa). Meanwhile, as expected women who report engaging in risky sexual behaviors are more likely to have HIV.

Finally, we compared the mean scale scores for HIV positive and negative women on a set of indicators for HIV knowledge, premarital sexual experience, and three indicators of a woman’s power in her personal relationships, where higher values reflect higher power (Table 2). The results suggest that HIV positive women have higher levels of knowledge of HIV transmission and prevention, more years of premarital sexual experience, and higher scores on the power scales reflecting household decision-making. Interestingly, at the bivariate level, HIV status was not significantly associated with attitudes towards wife beating or women’s control over sexual decisions.

**Table 2:** Differences in Mean Scores by HIV status, Women Respondents, 2004 CDHS.

	Mean Scale Score			
Index	HIV negativerespondents	HIV positiverespondents	*t*	*Sign.*
HIV Knowledge Scale *(range 0–15)* *N = 5,131*	7.06	7.69	−4.29	***
Years of Premarital Sexual Experience *(range 0–29)* *N = 5,144*	1.83	3.42	−6.07	***
Index of Household Decision-Making Power *(range 0–5)* *N = 5,145*	1.78	2.58	−8.45	***
Index for Opposition to Wife-Beating *(range 0–5)* *N = 5,150*	3.47	3.44	0.35	*n.s.*
Index for Attitudes toward Sexual Decisions *(range 0–4)* *N = 4,547*	3.31	3.38	−1.61	*n.s.*

*n* of women HIV positive = 349.

*** = p<0.001 in *t*-test.

#### Relationship between SES and Access to Health Care, Knowledge, and Sexual Behaviors

Overall, as expected, there was a positive and highly significant association between SES and access to health care (Table 3). Wealthier and more educated women were more likely to have visited a health facility in the last 12 months (57 and 66%, respectively), and to have heard of and visited a Voluntary Counseling and Testing Center (5 and 8%, respectively). Higher SES women were also significantly less likely to report barriers to accessing health care. In fact each increase in a woman’s educational attainment was associated with a decrease in the number reporting barriers to health care. Access to health care also varied by the different occupational categories. Women in professional or white collar professions were more likely to report visiting a CPDV (14%), more likely to have accessed a health facility within the last year, and faced the fewest barriers to health care of any occupational group.

**Table 3:** Relationship between SES and Access to Health Care, Women Respondents, 2004 CDHS.

	% Access CPDV			Visited Health Facility	Barriers to Health			
Respondent’s SES	NH	H	B	% Yes	0	1	2	3
**Wealth**								
Low	91	9	1	47	39	12	33	16
Medium	81	17	2	51	52	14	26	8
High	57	38	5	57	65	16	15	5
* χ^2^*		*618.4* ***		*35.3* ***		*407.3* ***		
* Gamma*		*0.609* ***		*0.136* ***		−*0.357* ***		
**Education**								
No Education	97	1	3	44	41	11	26	22
Primary	84	14	2	52	52	14	26	8
Secondary	54	41	5	57	59	15	21	5
Higher	26	67	8	66	65	15	21	3
* χ^ 2^*		*909.9* ***		*48.8* ***		*273.4* ***		
* Gamma*		*0.713* ***		*0.156* ***		−*0.235* ***		
**Occupation**								
Unemployed	71	26	3	47	57	15	20	9
Ag Sector	88	11	1	52	43	12	31	13
Manual/Domestic	68	28	4	58	59	15	20	7
Prof/White Collar	30	56	14	72	66	16	15	3
* χ^ 2^*		*472.3* ***		*71.3* ***		*175.1* ***		
* Cramer’s V*		*0.214* ***		*0.118* ***		*0.107* ***		

Note: NH = Not Heard; H = Heard but not been; B = Been.

χ^ 2^ = Chi Square; Gamma used for ordinal variables; Cramer’s V for nominal variables.

***p<.001.

A woman’s SES was also significantly associated with both protective and risky sexual behaviors, though the pattern was not consistent across different measures (Table 4). Condom use was significantly and positively associated with a woman’s SES. Specifically, women in high wealth households, with secondary or post-secondary education, and with professional/white collar jobs report dramatically higher rates of past and present condom use than women in the other SES groups. By contrast, condom use among women with no formal education or living in low wealth households was almost non-existent.

**Table 4:** Relationship between SES and Sexual Behaviors, Women Respondents, CDHS 2004.

	Protective Behaviors			Risky Behaviors			
	Condom UseEver (yes)	Condom UseLast Sex (yes)	Condom UseCurrently (yes)	Alcohol LastSex (any)	Sex OutsideRelation-ship(Yes)	Multiple Partners(yes)	Early First Sexualexperience (under 15)
*percent reporting behavior*							
Wealth							
Low	15.4	4.4	2.8	16.1	5.0	4.4	40.6
Medium	32.1	9.1	6.2	14.7	10.7	7.0	36.3
High	49.3	18.2	16.6	13.7	12.6	8.5	29.6
χ^ 2^	509.9***	191.7**	240.8***	16.0**	68.2***	26.8***	53.8***
Gamma	0.531***	0.513***	0.616***	−0.083***	0.313***	0.240***	−0.176***
Education							
No Educ.	1.6	0.9	0.1	11.6	1.0	0.4	45.0
Primary	27.5	8.0	5.8	16.6	9.1	7.2	36.2
Secondary	55.4	19.4	16.9	14.8	14.4	9.4	28.9
Higher	66.3	33.3	35.4	7.3	11.5	8.3	16.8
χ^ 2^	956.9***	298.5***	339.3***	59.3***	138.5***	87.2***	95.6***
Gamma	0.711***	0.621***	0.693***	−0.059**	0.455***	0.400***	−0.228***
Occupation							
Unemployed	36.8	14.8	12.9	8.7	9.6	6.9	43.8
Ag Sector	18.5	4.2	2.7	19.4	5.2	4.4	33.3
Man/Dom	43.8	14.4	11.5	17.0	14.5	9.2	27.9
Prof/Wcollar	68.2	20.2	20.5	18.5	17.1	9.0	9.4
χ^ 2^	365.9***	140.7***	161.9***	237.7***	86.9***	28.6***	158.8***
Cramer’s V	0.267***	0.167**	0.178***	0.152***	0.130***	0.075***	0.176***

χ^2^ = Chi Square.

**p<.01.

***p<.001.

The pattern is somewhat different for risky behaviors. Women in higher wealth households and post-secondary education reported less alcohol use at last sexual intercourse and were less likely to initiate sexual activity before the age of 15. However, these high SES women were the most likely to report recent sex with a partner other than their current partner, and to have had more than 1 sexual partner over the previous year.

A similarly complex story can be told of the relationship between occupation and the incidence of risky sexual practices. The relationship between drinking alcohol and sex was most prevalent among agricultural workers and professional/white collar occupations, while unemployed women were the least likely to report this behavior. Meanwhile, unemployed women were the most likely to report early sexual activity, and women working in the agricultural sector were least likely to report sex outside of relationships or multiple partners, by contrast, both professional workers and manual/domestic workers had notably higher rates of these practices.

Finally, we compared mean scores on the knowledge, premarital sex, and decision-making power scales among women in different SES groups (Table 5). As expected, higher SES women reported consistently higher levels of knowledge about HIV transmission and prevention methods. Women with secondary and post-secondary education had scores that were almost double that of women with no formal education. A woman’s occupation was also significantly associated with knowledge of HIV prevention method, with women in professional/white collar professions commanding the highest mean knowledge score and women in agricultural professions reporting the lowest scores. SES was also significantly and positively associated with the duration of premarital sex. Whether measured by wealth, education, and occupation, high SES women reported consistently greater gaps between their initiation of sexual activity and their first marriage.

**Table 5:** Relationships between SES and HIV Knowledge, Relationship Power, and Premarital Sexual Exposure, Women Respondents, CDHS 2004.

Indicators of Women’s Power					
SES	Knowledge of HIV &HIV PreventionMethods	Higher DomesticDecision MakingAuthority	Empowered FemaleSexual DecisionMaking	Opposition To WifeBeating	Years of PremaritalSexual Exposure
*mean index scale scores*					
All FemaleRespondents	7.10	1.84	3.30	3.47	1.93
Wealth					
Low	5.89	1.69	3.23	3.13	1.33
Medium	6.97	1.84	3.24	3.33	2.03
High	8.18	1.97	3.41	3.83	2.38
*F*	424.2	15.6	20.1	99.5	46.4
Significance	***	***	***	***	***
Education					
No Educ	4.87	1.50	3.06	3.04	0.51
Primary	6.80	1.95	3.30	3.30	1.97
Secondary	8.51	1.84	3.45	3.83	2.53
Higher	9.29	2.82	3.56	4.42	4.37
*F*	681.0	30.7	35.9	83.7	89.4
Significance	***	***	***	***	***
Occupation					
Unemployed	7.37	1.11	3.29	3.59	1.56
Ag Sector	6.26	2.09	3.26	3.22	1.80
Man/Dom	7.56	2.36	3.35	3.46	2.29
Prof/Wcollar	9.02	3.32	3.48	4.42	4.19
*F*	159.0	299.0	4.8	42.3	33.9
Significance	***	***	**	***	***

N = 5154.

ANOVA F-test significance levels: ** = p<0.01; *** = p<0.001.

Higher SES women also report generally higher levels of decision-making power and authority in their relationships (Table 5). Women in the highest wealth category had the highest score on a domestic decision-making control index and both indicators of attitudes toward women’s power in relationships (the ability to refuse sex and opposition to wife beating). Educational attainment and decision-making power was somewhat more complicated. As expected, women with post-secondary education had the highest score on all three indicators of power; however, women with secondary education had a slightly lower mean score on domestic decision-making authority compared to women with primary level education. Post-hoc significance tests suggest that the differences between these two intermediate groups were not statistically meaningful. Finally, decision-making power was significantly associated with a respondent’s occupation. Women in professional/white collar professions commanded the highest average decision-making authority on all three indicators compared to the other professions. Women in agriculture and manual or domestic occupations generally expressed the weakest attitudes toward a woman’s ability to refuse sex or justifications for wife beating. Women who were unemployed had the lowest levels of household decision-making authority, but intermediate attitudes towards control over sexual decisions and wife beating.

#### Relationship between Access to Health Care, HIV Knowledge, and Sexual Behaviors

Because access to health care and HIV knowledge appear to be positively related to HIV status among women in Cameroon, we explored whether or not people who had better access to health care resources and higher knowledge of HIV adopted safer sexual practices (Table 6). On the one hand, access to health care was associated with increased protective sexual behaviors. Women who have visited CPDV or general health clinics are more likely to report condom use. Similarly, women with higher scores on the HIV knowledge scale reported consistently higher condom use.

**Table 6:** Relationship between Access to Health Care, Knowledge, and Sexual Behaviors, for Women Only, CDHS 2004.

	Protective Behaviors			Risky Behaviors		
	Condom Use Ever	Condom Use Last Sex	Condom Use Currently	Alcohol Last Sex	Sex Outside Relationship	Multiple Partners
*percent reporting behavior*						
Visited Health Clinic						
NO	25.3	10.8	47.5	12.9	8.2	6.9
YES	41.0	11.8	52.5	16.5	10.7	6.5
*χ^ 2^*	*141.4* ***	*1.1*	*0.7*	*84.2* ***	*9.6* **	*0.2*
*Cramer’s V*	*0.166* ***	*0.015*	*0.012*	*0.128* ***	*0.043* **	*0.007*
Visited CPDV Clinic						
NO	32.8	9.1	11.1	14.8	9.4	6.6
YES	57.4	20.8	18.3	15.8	11.7	8.3
*χ^ 2^*	*39.1* ***	*7.1* **	*23.4* ***	*1.7*	*0.9*	*0.6*
*Cramer’s V*	*0.087* ***	*0.037* **	*0.067* ***	*0.018*	*0.013*	*0.011*
Score on HIV Knowledge Index						
Under 5	7.9	2.5	1.4	11.8	3.7	2.5
5–6	19.6	7.0	4.9	14.7	7.3	5.6
7	28.4	8.8	6.4	14.2	10.0	6.9
8	42.4	14.5	11.3	17.4	10.7	8.0
9	45.7	14.1	12.5	14.8	11.7	7.9
10 and up	53.3	19.7	18.6	15.3	13.4	9.0
*χ^ 2^*	*572.5* ***	*157.3* **	*187.8* ***	*15.5*	*60.0* ***	*36.9* ***
*Gamma*	*0.335* ***	*0.370* **	*0.191* ***	*0.042* *	*0.242* ***	*0.213* ***

*p<.05.

**p<.01.

***p<.001.

On the other hand, health care access is not systematically related to levels of risky sexual behaviors (sex outside of relationships, having multiple partners, or mixing sex with alcohol). Women who had accessed a health clinic in the last twelve months were more likely to have consumed alcohol at their last sexual intercourse and to be unfaithful to their partners. Meanwhile, visits to CPDV clinics were not related to any of the measures of risky behavior.

As expected, the amount of knowledge of HIV prevention methods a woman commanded was significantly and positively associated with protective sexual behaviors. Women who scored less than 5 (out of 15) and below on the knowledge scale had the lowest rates of past and current condom use. On the other hand, women who scored 10 and above on the knowledge scale had higher rates of both current and past condom use. However, women who scored higher on the knowledge scale also had higher rates of risky sexual behavior, with higher rates of sex outside of relationships as well as greater incidence of multiple sexual partners in the last twelve months.

### Multivariate analyses

To ascertain how the positive SES-HIV relationship observed at the bivariate level is mediated by key theoretically relevant and/or empirically significant indicators, we ran two multivariate binary logistic regression models to predict the likelihood of a woman testing positive for HIV in Cameroon (Table 7). The first (crude) model illustrates the effect of a woman’s SES characteristics on the odds of testing positive for HIV. The second (full) model includes indicators of the various intervening variables illustrated in our theoretical model above.

**Table 7:** Association between SES and Other Respondent Characteristics on the Odds of Testing Positive for HIV among Women in Cameroon, Multiple Logistic Regression Results for Crude and Full Models.

	OR_crude_	CI 95%	*Sign*	OR_full_	CI 95%	*Sign*
**Respondent Socioeconomic Status**						
Respondent’s Education						
No formal education (reference)						
Primary Education	1.591	(1.078–2.348)	**	1.068	(0.646–1.765)	
Secondary Education or Higher	1.692	(1.119–2.558)	**	1.073	(0.612–1.881)	
Respondent’s Occupation						
Agriculture (reference)						
Unemployed	0.736	(0.531–1.021)		1.040	(0.712–1.519)	
Manual or Domestic	1.283	(0.928–1.774)		1.082	(0.753–1.554)	
Professional/White Collar	0.773	(0.420–1.424)		0.473	(0.246–0.910)	*
Respondent’s Wealth Category						
Low (reference)						
Medium	1.852	(1.313–2.613)	***	1.398	(0.973–2.008)	
High	1.940	(1.362–2.763)	***	1.503	(0.993–2.275)	
**Partner’s Characteristics**						
Partner’s Education						
No Formal Education (reference)						
Primary Education				0.996	(.653–1.518)	
Secondary Education or Higher				0.907	(.594–1.386)	
Partner’s Occupation						
Agriculture (reference)						
Unemployed				0.623	(0.212–1.831)	
Professional/White Collar				1.717	(1.180–2.500)	**
Manual or Domestic				1.752	(1.182–2.596)	**
**Access to Health Care**						
Visited a Voluntary Counseling & Testing Center				1.852	(1.063–3.227)	*
Visited a Health Facility in the Last 12 Months				1.272	(0.991–1.633)	
Faced Any barriers to Accessing Health Facilities				0.706	(0.549–0.909)	**
**Knowledge of HIV Prevention Method**						
Index of Positive HIV Knowledge				1.022	(0.963–1.084)	
**Power in Relationships**						
Domestic Decision-Making Power				1.119	(1.029–1.219)	**
Negative Attitudes - Wife Beating				0.917	(0.849–0.989)	*
**Sexual Behavior**						
Protective Behavior						
Condom at Last Sexual Intercourse				0.755	(0.515–1.107)	
Risky Behavior						
Years of Premarital Sexual Exposure				1.085	(1.056–1.115)	***
Sex with Person Other than Partner				0.834	(0.525–1.323)	
>1 Sex Partner Last 12 Months				1.298	(0.828–2.035)	
Early Sexual Exposure (before age 15)				0.941	(0.713–1.243)	
**Marital Status**						
Currently Married (reference)						
Never Married				1.006	(0.551–1.838)	
Currently Cohabitating				2.248	(1.556–3.246)	***
Formerly Married				3.590	(2.539–5.076)	***
**Control Variables**						
Religion						
Catholic (reference)						
Protestant				1.078	(0.828–1.404)	
Muslim				1.018	(0.648–1.599)	
Other				0.570	(0.322–1.006)	
Place of Residence						
Rural Residence				1.011	(0.733–1.394)	
Region						
Centre/Sud/Est (reference)						
Littoral/Sud Ouest				1.247	(0.859–1.810)	
Ouest/Nord Ouest				1.279	(0.811–1.858)	
Nord/Adamaoua/Extreme Nord				1.057	(0.656–1.704)	
Yaoundé				1.237	(0.779–1.963)	
Douala				0.611	(0.367–1.017)	
FIT STATISTICS						
Neg 2 LL	2337.1			2129.4		
Omnibus test of model coefficients	0.000			0.000		
Cox & Snell R2	0.011			0.252		
Nagelkerke R2	0.030			0.136		
H&L Test	3.101			8.201		
H&L Sig.	0.928			0.414		
Percentage correctly classified	93.4			93.4		

N = 4,916.

* = p<.05.

** = p<.01.

*** = p<.001.

Results of the crude model highlight the positive association between a woman’s education and household wealth and the prevalence of HIV in Cameroon. Women with higher levels of formal education and women living in high wealth households were nearly twice as likely to test positive for HIV as women with no formal education or who live in low wealth households. None of the occupational measures were statistically significant predictors of HIV status.

Results of the full model indicate that the significant association between a woman’s SES and her HIV status is attenuated when we account for other factors. After accounting for partners’ characteristics, access to health care, HIV knowledge, and power in relationships, neither education nor wealth are significant predictors of testing positive for HIV. An interesting exception in the full model is the newly significant coefficient for women in with professional/white collar occupations, who have a 53 percent lower chance of testing positive for HIV than women working in agriculture, net the impact of the other variables in the model (OR = 0.473). In the full model, one coefficient for partner’s occupation was significant; women whose partners had professional/white collar or manual/domestic jobs were roughly twice as likely to have tested positive for HIV as women whose partners worked in the agricultural sector.

Indicators for access to health care provided inconsistent results in that visits to a CPDV clinic was associated with higher odds of HIV infection, while reported barriers to accessing health care facilities was associated with lower odds of testing positive for HIV. The coefficient measuring visits to any health facility was positively related to HIV status, but fell just below the threshold of statistical significance. Interestingly, in the full model, the indicator of knowledge about HIV prevention methods was not a significant predictor of HIV status.

Two measures of women’s power were included in the full model and produced contradictory results (the measure for justifications for refusing sex was dropped in the multivariate model because of problems with missing data on over 500 cases). Women who had more authority over household domestic decisions had an elevated HIV risk (OR = 1.119), while those who were less tolerant of wife beating had a lower HIV risk as they were 9 percent less likely to test positive for HIV (OR = 0.917).

The measures of protective and risky behaviors were generally not significant predictors of HIV status (net the effects of other variables in the full model). For women in Cameroon, each additional year of premarital sexual exposure increased their odds of testing positive for HIV by 9 percent (OR = 1.085). Meanwhile, two marital status variables significantly increased the risk of HIV for Cameroonian women: relative to women who were currently married, those who were cohabiting were 2.3 times more likely and those who were widowed, divorced or separated were 3.6 times more likely to test positive for HIV (OR = 2.248 and 3.590, respectively). None of the control variables achieved the criterion for statistical significance.

## Discussion

The positive relationship between SES and rates of HIV infection in Cameroon at the bivariate level reflects a situation where increased access to health care, HIV information, and personal power (all associated with rising SES) have not yet translated into reductions in risky behaviors that are known to increase transmission of HIV. Higher SES women do have greater access to medical help, command greater knowledge of HIV prevention methods, report more use of condoms (albeit still relatively low), and express greater decision making authority within their relationships, but these fail to protect them from having the highest HIV rates among women in the country. Our multivariate analyses show that inclusion of measures of intervening factors attenuates the positive relationship between SES and HIV among Cameroonian women significantly. The SES-HIV relationship is influenced by partners’ occupation, marital status, length of premarital exposure, and power within relationships, and greater access to health care.

Given these results, the key question remains: *Why are the benefits of increased resources associated with higher SES not protecting women in Cameroon from HIV infection?* At least through 2004, any benefit that may have accrued from high SES appears to be offset by a tendency to engage in more risky sexual practices – including longer periods of premarital sexual activity, greater numbers of recent sexual partners, and higher rates of sex outside of relationships. This suggests that economic vulnerability and traditional gender roles may not be the only risk factors that put women in SSA at greater risk of contracting HIV. High SES women, in particular, may also be at increased risk through their relationships with higher SES men (who have been shown in previous research to engage in riskier practices [16]). These results support the idea that the pursuit of modern identities can create aspirations and desires for social mobility that can lead to riskier behaviors among high SES women [5], [17]. Conversely, high SES women could also be at risk not only from their primary partners (which we know is positively related to their HIV status) but from having concurrent relationships even within the context of marriage. As a recent analysis of DHS suggests, contrary to what was commonly presumed, it is women rather than men that are infecting their partners within relationships [13].

The results fail to support the expectations of traditional gender theory in that greater household and sexual decision-making power (and progressive attitudes toward wife beating) have not necessarily translated into lower risks of HIV in Cameroon. The unexpected positive relationship between ‘power’ and HIV status may reflect the spurious effects of a third common variable associated with socioeconomic status (perhaps culturally-driven riskier sexual behaviors among high SES males and females). It is also worth noting that delayed marriage and longer periods of premarital sexual activity significantly increases risk for high SES women [37]. Similarly, Fortson [38], in a multicounty analysis including Cameroon found that women with greater than 6 years of schooling were 16 percent times more likely to have had premarital sex, compare to women with more schooling.

With regards to results from our multivariate analysis, the attenuation of the SES-HIV relationship suggests that there may be no direct association between SES and HIV among women in Cameroon. This result mirrors findings from other studies in SSA [19], [25], [39]. It is possible this result reflects the complex intervening effects of the other variables associated with higher SES status among Cameroonian women, especially sexual behaviors and personal power [40]. As Gillespie et al. [39], note, it is also possible that the preexisting sexual behaviors of the wealthy at this early stage of the epidemic increased their vulnerability to the disease. The recently released 2011 CDHS will provide a great opportunity to assess if the association between SES and HIV changes as the epidemic matures.

Our study has several limitations. The cross-sectional nature of the data makes it difficult to determine the causal order of statistically significant relationships (i.e. did the visit to health facility or CPDV happen before or after the diagnoses of HIV infection). It is likely that women were more inclined to visit a health facility because they are feeling ill, which could reflect the fact that they had already contracted the disease. Similarly, women who already contracted HIV may be more likely to develop better knowledge of the disease (and of methods to prevent transmission), which could explain the positive bivariate relationship between HIV status and HIV knowledge.

Other potential limitations include the point that several key variables (such as risky sexual behaviors) are self-reported. Many researchers have argued that women in Sub-Saharan Africa usually under report either their premarital or their extramarital sexual activity [41]. Findings using self-reported data may be biased to the extent that sex outside of relationships, total number of partners, and condom use are misreported by women in a pattern that varies across the different SES groups [42]. It is worth noting that the risk associated with sexual relations with a person other than a partner can be moderated by whether a condom was used during sexual intercourse, which was not measured in this study. Similarly, we are unable to determine from the DHS data if the origin of HIV was from a spouse or regular partner (whose SES is measured), or from another sexual partner (whose SES is not available). We also have no direct measures of a woman’s sexual networks. Finally, future analyses would benefit from including direct measures for partner’s sexual behavior, women’s sexual networks, and direct exposure to gender violence, which have been critical factors in explaining higher rates of HIV among SSA women in other studies [16], [43]–[44].

## Conclusion

Taken as a whole, the results suggest that traditional approaches to HIV prevention which rely on poverty reduction, improving access to health care, improving HIV knowledge, and boosting women’s social and economic power may be insufficient to address key drivers of HIV infection among women in Cameroon. In particular, the persistence of risky behaviors among higher SES populations suggests that a targeted program to change cultural practices and mores might be required to prevent the continued spread of the disease. This study adds to the growing body of work which notes that in certain areas poverty may not be the main driver of HIV risk, and that pathways between SES and HIV infection can vary by SES group [5], [19], [39]. Targeted interventions to address the impacts of sex outside of relationships and reducing sexual concurrency among higher SES women would appear to be warranted.
